# Integration of TLS and HLS Data for Non-Destructive Structural Damage Assessment of Building Structures: A Case Study of a Small Hydropower Plant

**DOI:** 10.3390/ma18235352

**Published:** 2025-11-27

**Authors:** Piotr Kędziorski, Jacek Katzer, Marcin Jagoda

**Affiliations:** 1 Faculty of Civil Engineering, Environmental and Geodetic Sciences, Koszalin University of Technology, Śniadeckich 2, 75-453 Koszalin, Poland; piotr.kedziorski@tu.koszalin.pl; 2 Faculty of Geoengineering, University of Warmia and Mazury in Olsztyn, Prawocheńskiego 15, 10-720 Olsztyn, Poland; jacek.katzer@uwm.edu.pl

**Keywords:** non-destructive measurement, handheld laser scanning (HLS), terrestrial laser scanning (TLS), 3D scanning, point cloud integration, crack detection

## Abstract

**Highlights:**

**What are the main findings?**

- TLS data alone was insufficient to capture the full geometry of damage zones.
- TLS and HLS IR data integrated to fill measurement gaps in dam structure.
- Both HLS modes yielded consistent crack widths with ≤0.37 mm difference.

**What are the implications of the main findings?**

- Integration fills TLS blind spots and increases diagnostic completeness.
- Precise geometry improves structural risk assessment and decision-making.
- The method supports early detection and monitoring in hydraulic infrastructure.

**Abstract:**

This study demonstrates the effectiveness of integrating terrestrial laser scanning (TLS) and handheld laser scanning (HLS) for structural diagnostics. The research was conducted on a Small Hydropower Plant (SHP) in Koszalin, Poland. TLS was used to capture the general geometry of the object, while HLS operating in infrared (IR) and blue light modes enabled high-resolution documentation of local damage. Areas of interest were identified using the Surface Variation parameter, and selected zones were scanned with HLS. Both HLS modes delivered consistent results, with differences not exceeding ±0.37 mm. The IR mode proved particularly useful in constrained spaces, allowing for precise measurements without the use of reference markers. Comparative analyses of cross-sections through a major crack confirmed that both HLS modes produce repeatable results with submillimeter accuracy. Integrating TLS and HLS data resolved blind spots inherent to TLS and produced a complete point cloud preserving both global geometry and local detail. The findings confirm the applicability of this hybrid approach in assessing structural damage and highlight its relevance in civil engineering applications. The proposed workflow is effective for documenting inaccessible or complex geometries while optimizing data volume and acquisition time (R1-C10).

## 1. Introduction

In recent years, 3D scanning techniques have become an indispensable tool in diagnosing the technical condition of buildings and civil engineering structures, including hydraulic structures. Terrestrial Laser Scanning (TLS) enables rapid and precise mapping of large-scale object geometry, providing high-resolution and accurate data [1,2,3]. The application of TLS in documenting hydraulic structures, such as dams, channels, or hydroelectric power plants, has been described in the literature [4,5,6], demonstrating its usefulness for both inventory purposes and structural deformation analysis. Continuous monitoring of the technical condition of building structures is a critical aspect of their operational lifecycle. Every structure has a limited lifespan, and its ongoing monitoring, whether through direct methods or contact-based devices (e.g., crack gauges), can be both time-consuming and labor intensive (thus costly) and may not always provide comprehensive information about the object’s condition. In this context, TLS offers an attractive alternative, enabling rapid, non-contact, and high-resolution acquisition of object geometry data [1,7,8,9,10,11,12,13]. However, despite its high precision and flexibility, TLS has limitations. Acquiring “dense” and precise information about an entire object is time-consuming, and the resulting data volume is often excessive, leading to overrepresentation in certain areas of the measured object. Additionally, blind spots and incomplete data coverage in hard-to-reach areas are common issues with this technology [11,12,13]. To address these gaps, photogrammetry [14,15,16] or low-cost LiDAR scanners [17,18,19] are often employed.

An alternative solution, not yet sufficiently explored in the context of buildings and civil engineering structures, is the use of precise handheld scanners (HLS). These devices offer extremely high measurement accuracy (up to ±0.02 mm), great mobility, and the ability to measure in hard-to-reach spaces [20,21]. Their effectiveness has been demonstrated in many scientific fields, like documenting cultural heritage artifacts [22,23,24], in medicine [25,26,27], agriculture [28,29], and material research [30]. However, their use in hydraulic engineering remains largely unexplored. The presented research program focuses on integrating TLS with HLS to achieve detailed mapping of local damage while preserving the geometry of the entire object. This approach allows TLS measurements to be conducted with lower point density, reducing the time required for measurement and data processing. At the same time, it retains detailed information about areas of interest (e.g., cavities, cracks, or fissures), which are measured using HLS and integrated with TLS data. This approach enables not only localized assessment of an object’s condition but also a comprehensive analysis, allowing damage to be located in relation to the entire structure. This facilitates early detection of geometric changes, their monitoring over time, and informed engineering decisions regarding repairs, usage restrictions, or decommissioning. Such an approach is particularly critical for hydraulic infrastructure, where damage can pose significant risks to the environment and public safety [31,32]. The presented case study of the Small Hydropower Plant (SHP) in Koszalin (Poland) evaluates the practical potential of this approach in real-world conditions. The aim of this study is to present the possibilities of integrating data from TLS and HLS, assess their quality, and highlight the benefits of a hybrid approach in the technical diagnostics of structures. Assessment of the potential for full quasi-automation of the full measurement process was also considered and discussed.

## 2. Measured Object and Used Tools

**Figure 1:**
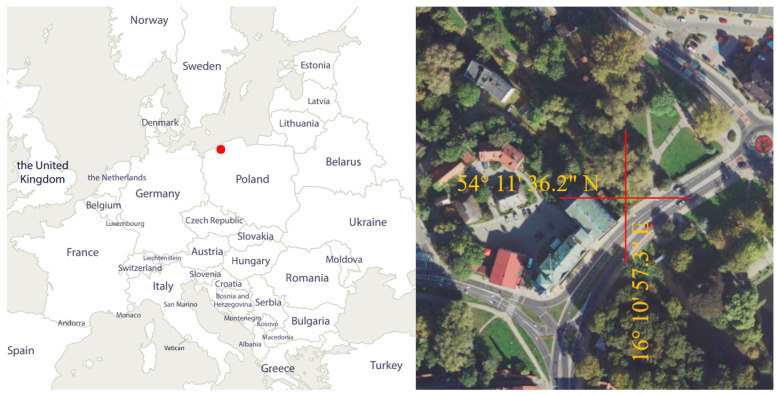
Location of the SHP.

The object of the analysis is an SHP located at the site of a historic water mill. The current form of the complex dates back to 1838–1842, and it has functioned as a hydropower plant since 1924. After the post-war period, the mill building was adapted for museum purposes, while the dam was repurposed to restart the SHP. The plant is equipped with three pump turbines, each with a capacity of 45 kW, operating at an average flow of 1 m^3^/s and a 6 m head [33,34,35]. The location of the SHP is shown in Figure 1.

**Figure 2:**
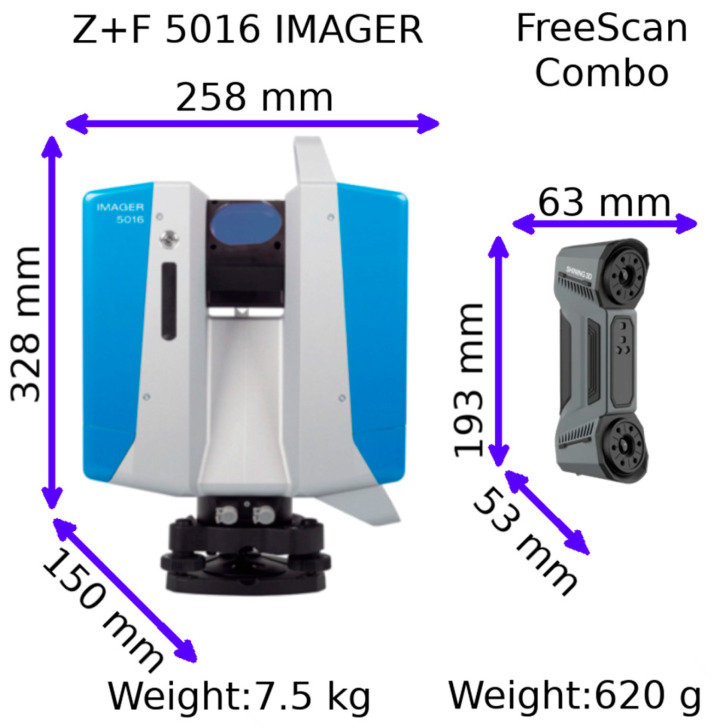
Comparison of size and weight of used TLS and HLS.

The measurement of the power plant was performed using a Z+F Imager 5016 phase-based TLS (Zoller & Fröhlich, Wangen im Allgäu, Germany) and the FreeScan Combo HLS (Shining 3D, Hangzhou, China). Both devices, their sizes and weights are shown in Figure 2.

The Z+F Imager 5016 enables data acquisition within a range of 0.3–365 m, with a maximum speed of 1.1 million points per second. The accuracy is specified as ≤1 mm ± 10 ppm, which refers to the positional accuracy of each measured point. (R2-C1) The scanning range is 360° (horizontal) and 320° (vertical). The second device is the 620 g FreeScan Combo, which has two operating modes: blue structured light (Blue), with an accuracy of up to ±0.02 mm, and infrared light (IR), with an accuracy of up to ±0.05 mm. Reference markers are required in Blue mode, while scenes are merged in real time without markers in IR mode using SLAM algorithms.

## 3. Workflow of the Research Program

**Figure 3:**
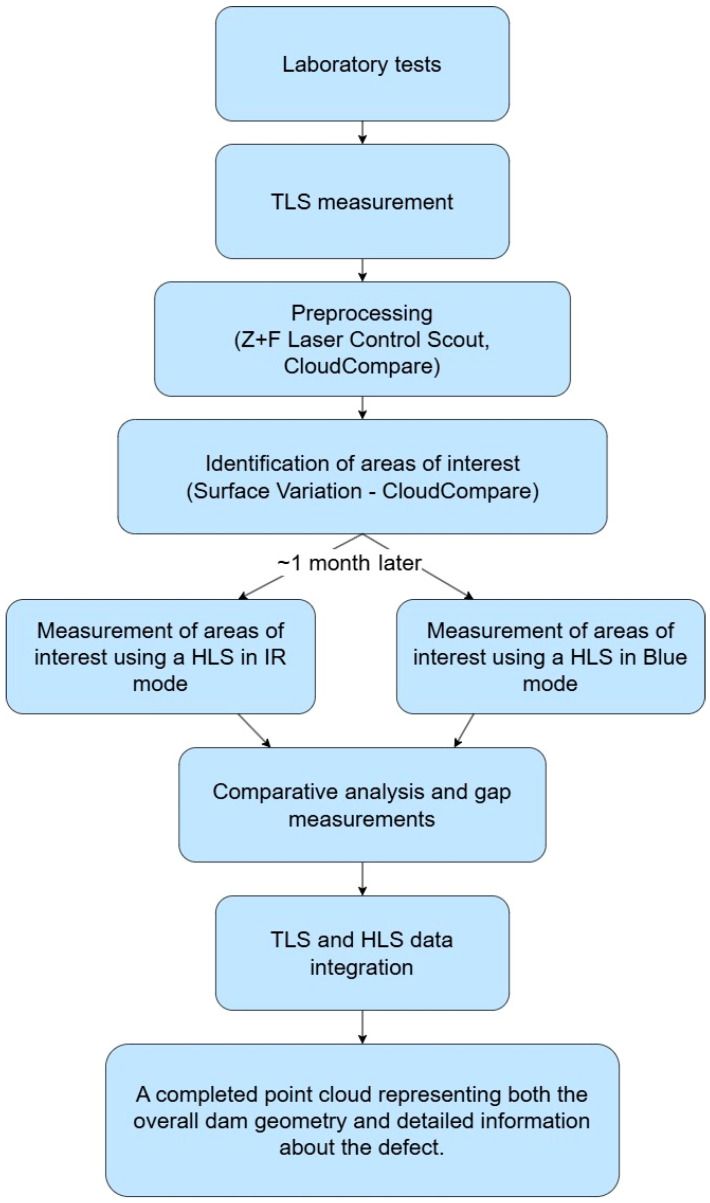
Workflow.

The research was divided into two parts: laboratory tests and measurements of a real building structure—a small hydropower plant. Figure 3 illustrates the complete process of data acquisition, analysis and integration.

**Figure 4:**
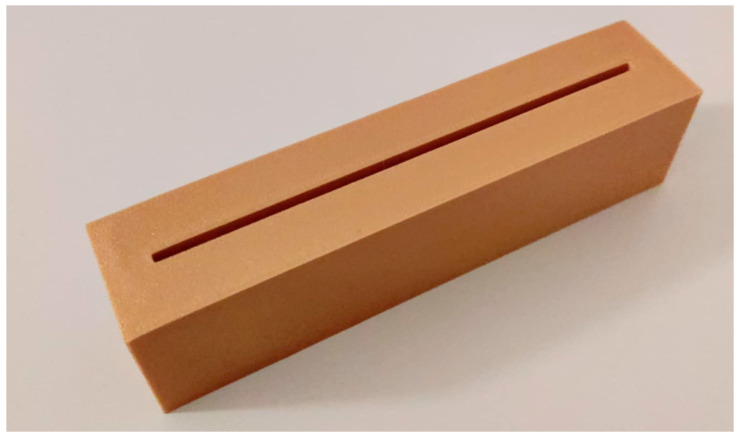
Three-dimensional print of a sample simulating a 2.5 mm crack.

The first stage of the research program involved laboratory measurements. The initial part included an independent evaluation of the Blue mode geometric accuracy. It was selected for further analysis because, according to the manufacturer’s technical data, it has the highest declared positioning accuracy of measurement points. Three additional reference models were prepared and printed using 3D technology for this purpose. Figure 4 shows an example of a printed model. These models simulated gaps with widths of 2.5, 5, and 7.5 mm and a length of 12 cm. Based on the measurements of these samples, cross-sections were created at three points (beginning, middle, and end) to verify the scanner’s precision against known nominal values.

**Figure 5:**
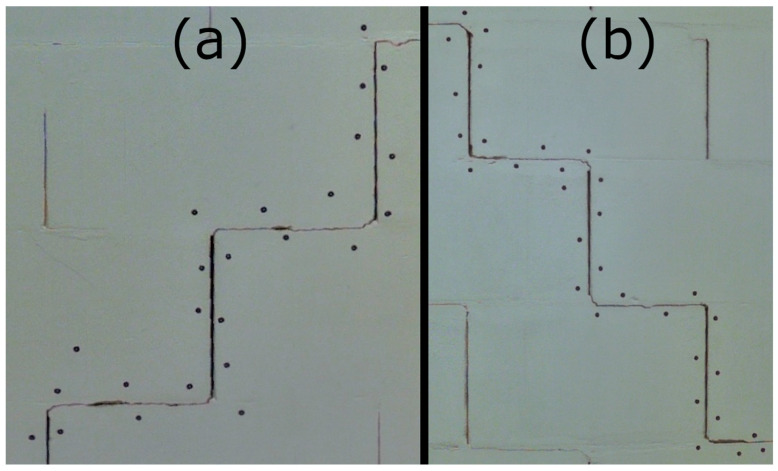
View of the first (**a**) and second (**b**) laboratory cracks.

In the second part of the laboratory tests, measurements were carried out to verify the applicability of the proposed workflow and test the feasibility of integrating TLS and HLS datasets. A test wall made of autoclaved aerated concrete (AAC) blocks was used to simulate structural defects in a controlled environment. Two artificial cracks were created in the wall to serve as reference features for evaluating the scanning and registration procedures. The view of the simulated cracks is shown in Figure 5. The wall was scanned using both a TLS and a HLS. The TLS instrument was positioned approximately 1.5 m from the wall and operated in medium density mode (6 mm spacing at 10 m). The HLS measurements were acquired with a point spacing parameter set to 0.2 mm for both Blue and IR modes. All datasets were postprocessed in CloudCompare (v. 2.13.1). Registration was performed using the Iterative Closest Point (ICP) algorithm (R1-C3, C1).

**Figure 6:**
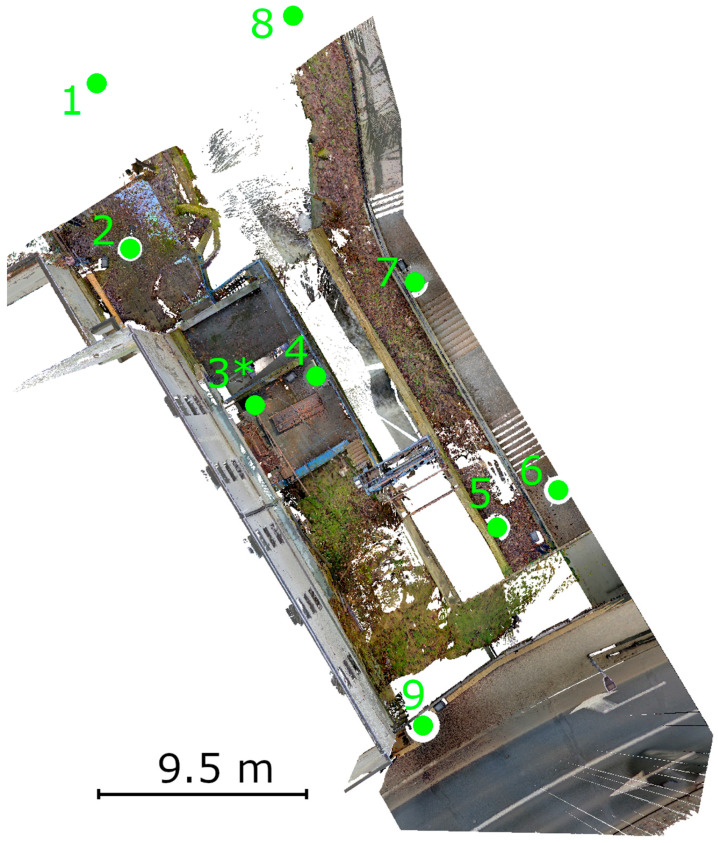
The location of the TLS measurement stations around the structure is shown below, *—station inside the machine room.

The second stage of the research program involved field measurements of a building structure—a small hydropower plant. Field measurements were carried out using nine TLS stations (medium density mode—6 mm spacing at 10 m): eight around the object and one inside the power plant building (machine room). Their layout is shown in Figure 6 (R2-C2). Initial data processing and registration were conducted using Z+F Laser Control Scout software (v. 9.1.0). A single scan contained an average of 170 million points. The data were exported to the E57 format and imported into CloudCompare for cleaning and data range reduction. The point clouds were merged and resampled at a 2 mm interval, reducing redundancy and resulting in a final point cloud of 168,026,884 points.

**Figure 7:**
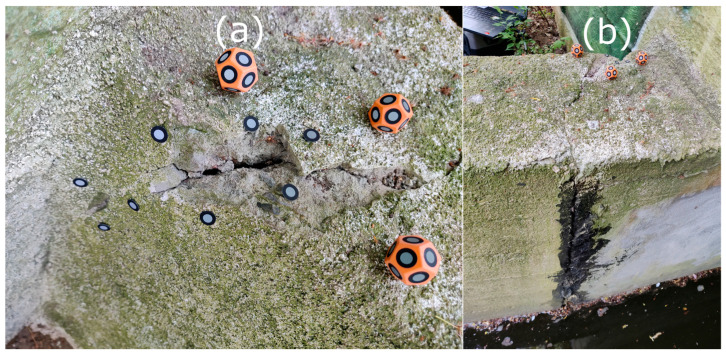
View of the defect identified during TLS measurement prepared for HLS measurement. (**a**) Smaller area measured using Blue mode. (**b**) View of the crack measured using IR mode.

After processing the TLS data, a defect in the power plant’s structure was identified on the 3D model, requiring a more precise measurement tool for detailed analysis. For this purpose, HLS was used. The SHP measurement of a defect in the power plant’s structure (see Figure 7) was conducted in both Blue and IR modes. In Blue mode, with a 0.1 mm point spacing, an area of approximately 0.10 m^2^ was scanned, resulting in a point cloud of 21,051,746 points. In IR mode (0.2 mm spacing), the same area, extended to include a portion of a crack on the water inflow side, was scanned, yielding 9,487,461 points over approximately 0.22 m^2^. The data was preprocessed in FreeScan software (v. 2.0.1.5) and exported in ASCII format. The point clouds were registered with the TLS data in CloudCompare and used for further analysis.

## 4. Results

### 4.1. Labolatory Test

**Figure 8:**
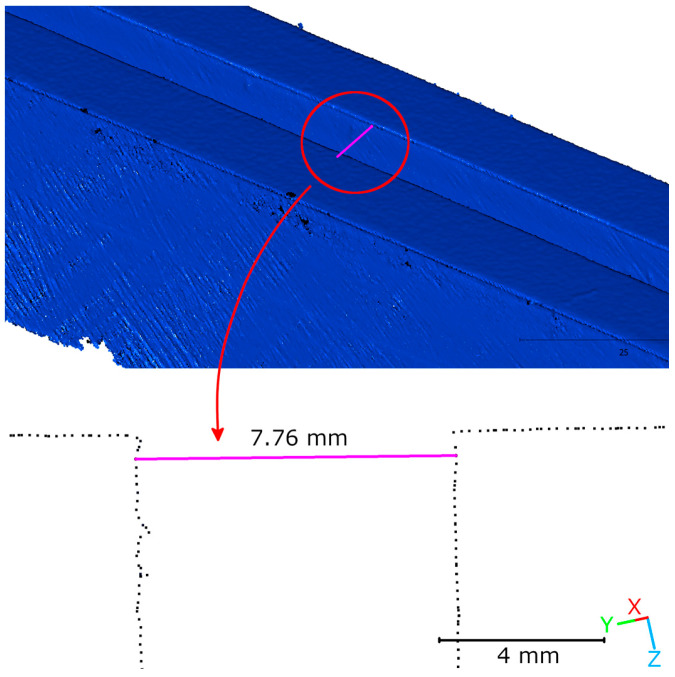
Measurement of an artificial defect using HLS in Blue mode for scanner accuracy assessment.

The results of the laboratory tests on the geometric accuracy of the HLS scanner in Blue mode were based on cross-sections. Table 1 summarizes the measurement results in relation to the model values, and Figure 8 shows an example of a cross-section visualization. For the smallest gap (2.5 mm), the average measured width was 2.67 mm, deviating +0.17 mm from the nominal value. For gaps measuring 5 and 7.5 mm, deviations of +0.11 and +0.22 mm, respectively, were recorded. All measurement series were characterized by a low standard deviation (0.04–0.05 mm), confirming the repeatability of the measurements. The results indicate that reproducing crack geometry with this technology allows for an accuracy of 0.2 mm (R1-C5).

**Figure 9:**
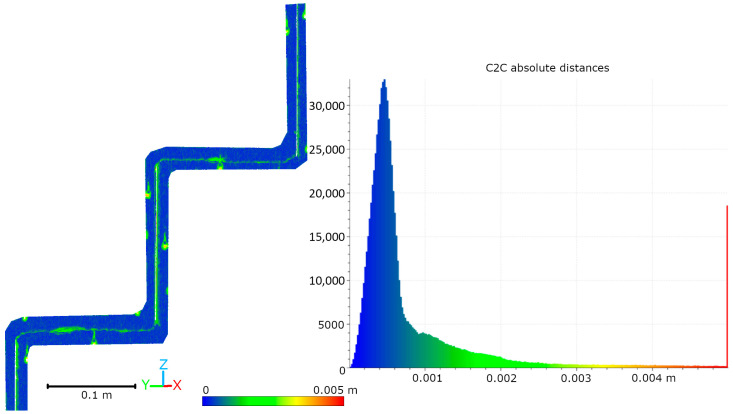
Cloud-to-cloud distance and distribution histogram between TLS and Blue data for Laboratory Crack 1.

**Figure 10:**
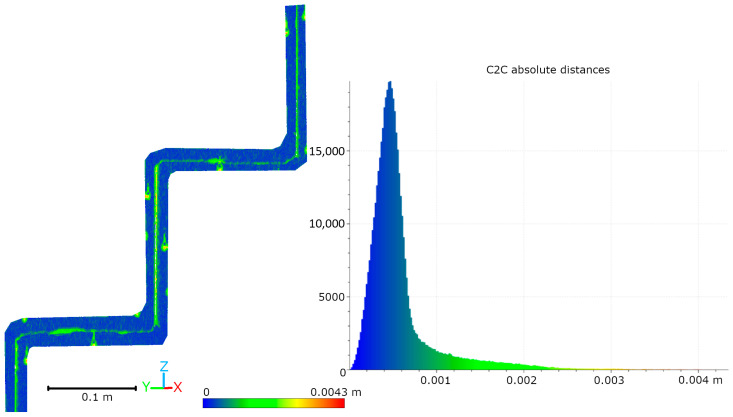
Cloud-to-cloud distance and distribution histogram between TLS and IR data for Laboratory Crack 1.

**Figure 11:**
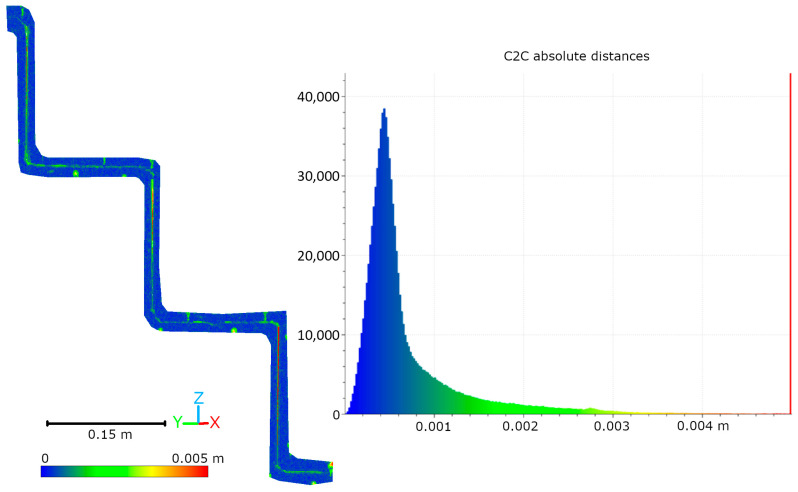
Cloud-to-cloud distance and distribution histogram between TLS and Blue data for Laboratory Crack 2.

**Figure 12:**
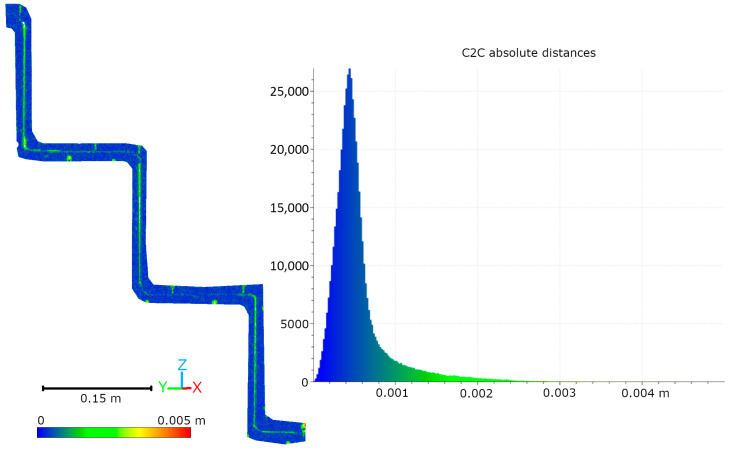
Cloud-to-cloud distance and distribution histogram between TLS and IR data for Laboratory Crack 2.

**Figure 13:**
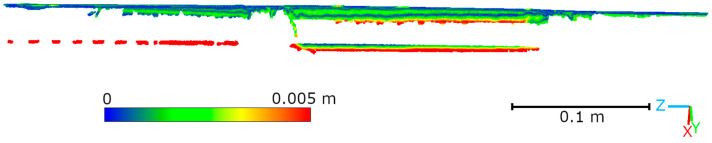
Visualization of Laboratory Crack 1 showing the ability of Blue mode to capture internal crack surfaces.

Subsequent laboratory analyses involved measuring simulated cracks. Registration between TLS and HLS point clouds was performed using the ICP algorithm. For Gap 1, the RMSE values were 1.08 mm (Blue mode) and 1.07 mm (IR mode) calculate on a 49,999 point sample. For Gap 2, the RMSE values were 0.92 mm and 0.93 mm, respectively. Distances between point clouds (TLS–Blue and TLS–IR) were calculated for fragments covering the immediate vicinity of two artificially created cracks. The number of points in the analyzed sections was as follows: for gap 1—23,456 (TLS), 489,577 (IR), 857,338 (Blue); for gap 2—29,907 (TLS), 547,196 (IR), 956,850 (Blue). The results are summarized in Figure 9, Figure 10, Figure 11 and Figure 12. The histograms of the differences indicate a distribution around 0.5 mm. When comparing TLS data with Blue mode data, an increase in the number of points with a deviation greater than 5 mm is visible. This phenomenon results from structural light’s greater ability to penetrate the crack. Blue mode not only recorded the wall surface and its crack, but also fragments of the interior of the gap, as illustrated by gap 1 (Figure 13). The deeper parts of the gap were not mapped in the case of TLS and IR data, resulting in greater consistency between these modes. In both cases, the greatest differences occur within the crack itself, suggesting that its geometry is mapped differently. The results confirm the possibility of precisely registering HLS data relative to TLS despite the significant difference in measurement data resolution.

**Figure 14:**
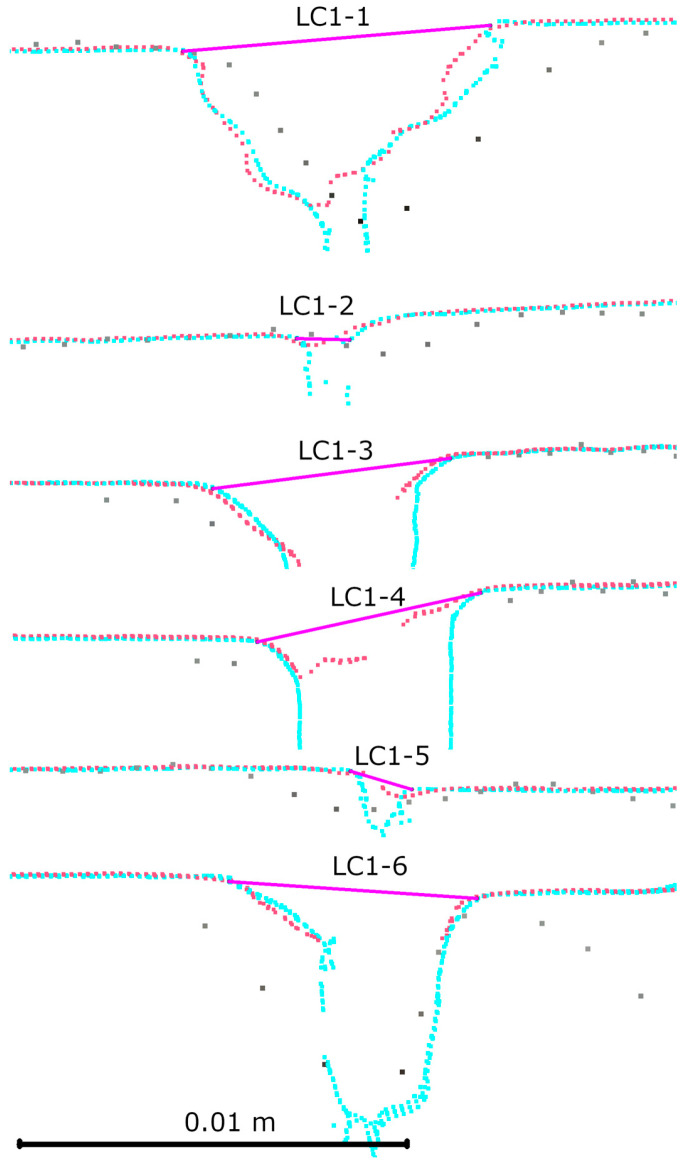
Cross-sections for first laboratory defect: gray—TLS, red—HLS IR mode, blue—HLS Blue mode.

**Figure 15:**
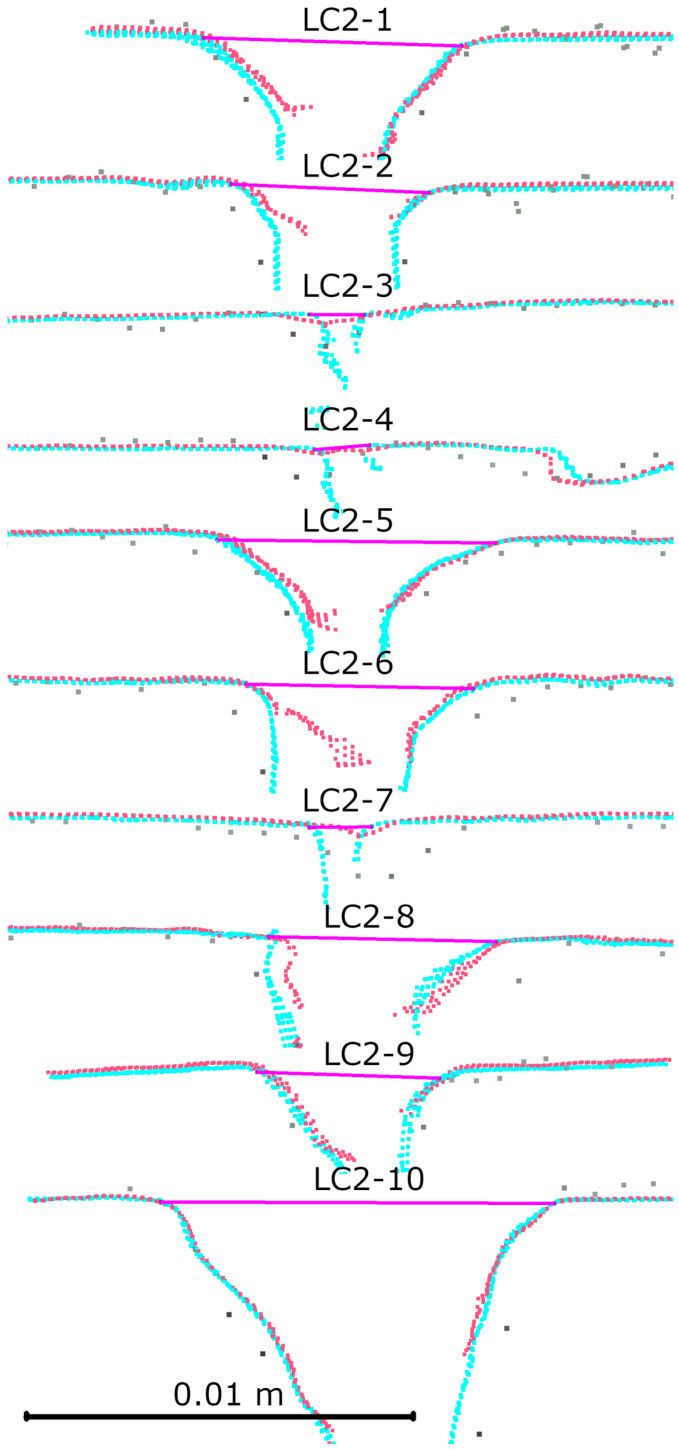
Cross-sections for second laboratory defect: gray—TLS, red—HLS IR mode, blue—HLS Blue mode.

After registration, an analysis of the laboratory crack cross-sections was performed. Six cross-sections were made for the first crack, evenly spaced along its entire length. The results are shown in Figure 14. Ten cross-sections were made for the second crack, also spaced along its entire length. The results are shown in Figure 15. The detailed results of the measurements are presented in Table 2. Upon analyzing both the graphical and tabular results, it was evident that some cracks could not be measured in IR mode. In this mode, the HLS scanner records narrow cracks with overly generalized geometry, making it impossible to determine where a given crack begins and ends. Only slight changes in geometry are visible (e.g., LC1-2, LC2-3, or LC2-7). The test results confirm this phenomenon for cracks of less than 2 mm. However, it is unclear up to what width this problem occurs. The problem does not occur in the case of cracks >5 mm (e.g., LC2-2, LC2-8). However, the group of cracks with a width of 2–5 mm was not present in the set of results, making it difficult to determine the minimum crack size that the IR mode can correctly handle. Upon analyzing the LC1-4 crack, we found that, despite its 6 mm width, we could not measure it correctly due to high noise levels in the cavity area. This is due to the angle of the inner edge of the crack relative to the wall plane, which is approximately 90 degrees. Similar noise effects can be seen in cross-sections LC2-6 and LC2-2 (R1-C7).

### 4.2. Measurement of the Small Hydropower Plant

**Figure 16:**
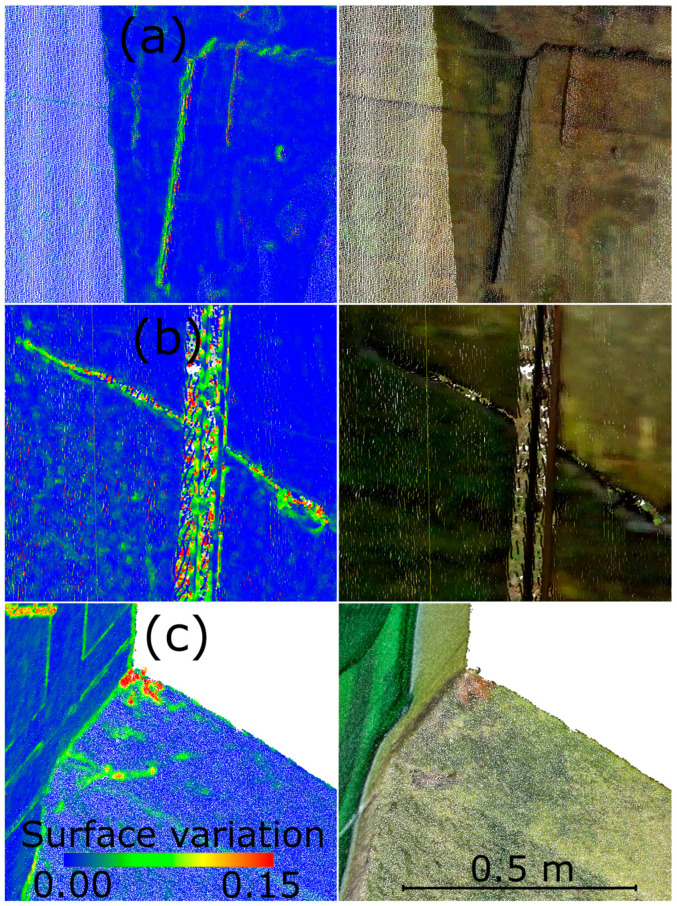
Defects detected using Surface Variation values. (**a**) Defect 1. (**b**) Defect 2. (**c**) Defect 3.

**Figure 17:**
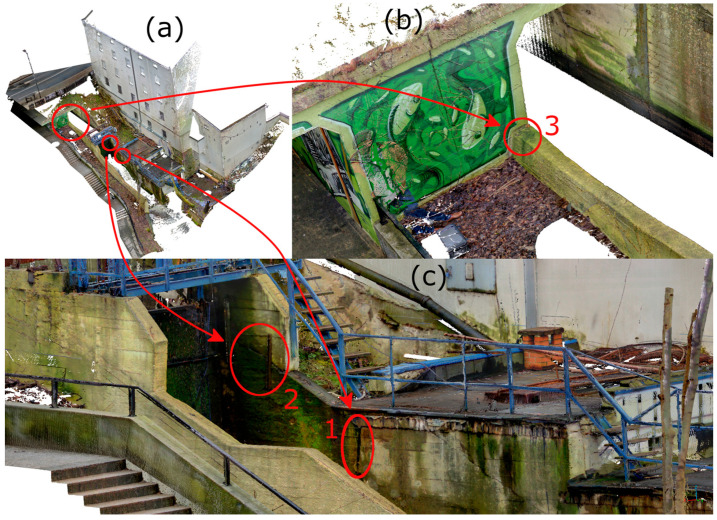
Location of defects against the point cloud representing the dam; (**a**) view of the entire point cloud; (**b**) detailed view of defect 3; (**c**) detailed view of defects 1 and 2.

After the field measurements were completed, the data was preliminarily processed using the scanner manufacturer’s software. The point clouds were colored and registered between stations. The final RMSE value for registration across all nine stations was 2.7 mm (R2-C4). Initial analyses was focused on identifying areas of interest. For the TLS point cloud, the Surface Variation geometric feature was calculated in CloudCompare with an analysis radius of 0.01 cm. Based on this, color-coded interpretations were created to visually highlight potential defects. The 0.01 m radius was empirically selected based on preliminary analyses to ensure adequate sensitivity to surface anomalies while limiting the impact of noise. Although no formal sensitivity analysis was performed, this value was adequate for the resolution and characteristics of the TLS data used in the study (R1-C4). The analysis identified areas of interest, shown in Figure 16, which were compared with the original point cloud. The detected cracks served as the basis for planning detailed HLS measurements. Considering both the Surface Variation analysis results and field constraints, one defect (no. 3) was selected for further investigation. Defects 1 and 2 were located directly above the outtake canal, which is in use. As a result, these areas were inaccessible for safe scanning. Because the object under study is a functioning hydroelectric power plant, safety and access limitations prevented close-range measurements in these regions (R1-C3). The location of the defects is shown in Figure 17.

**Figure 18:**
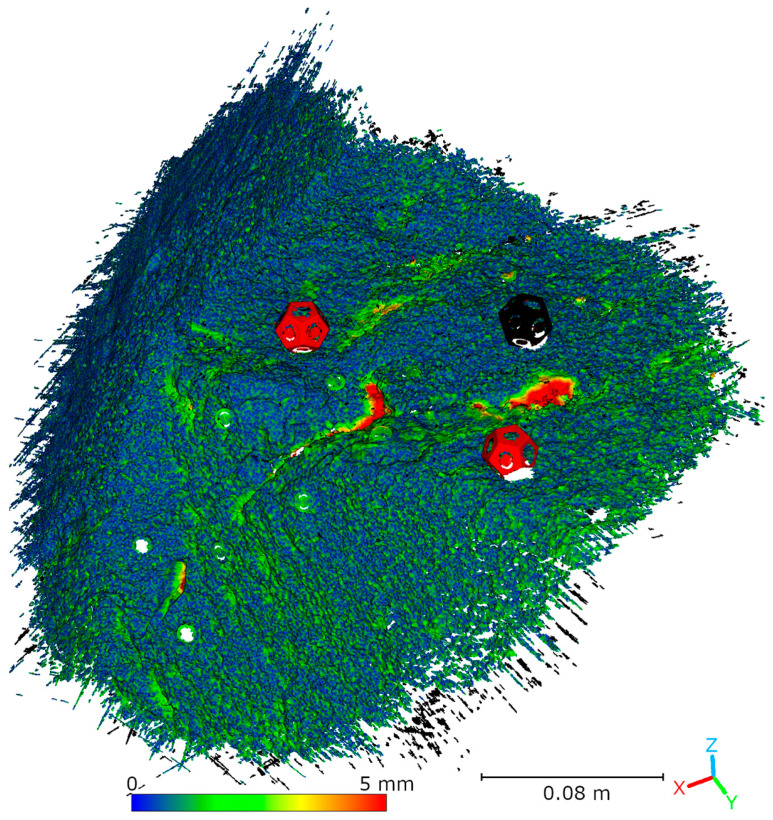
Comparison of TLS and HLS Blue scan via cloud-to-cloud distance (C2C).

**Figure 19:**
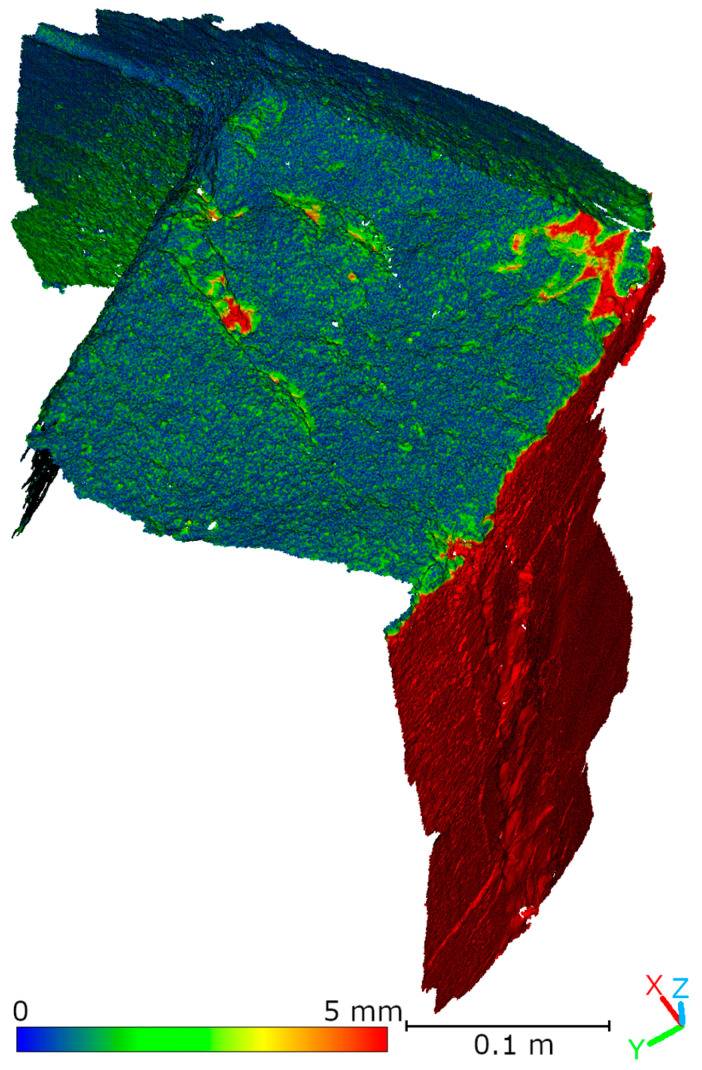
Comparison of TLS and HLS IR scan via cloud-to-cloud distance (C2C).

**Figure 20:**
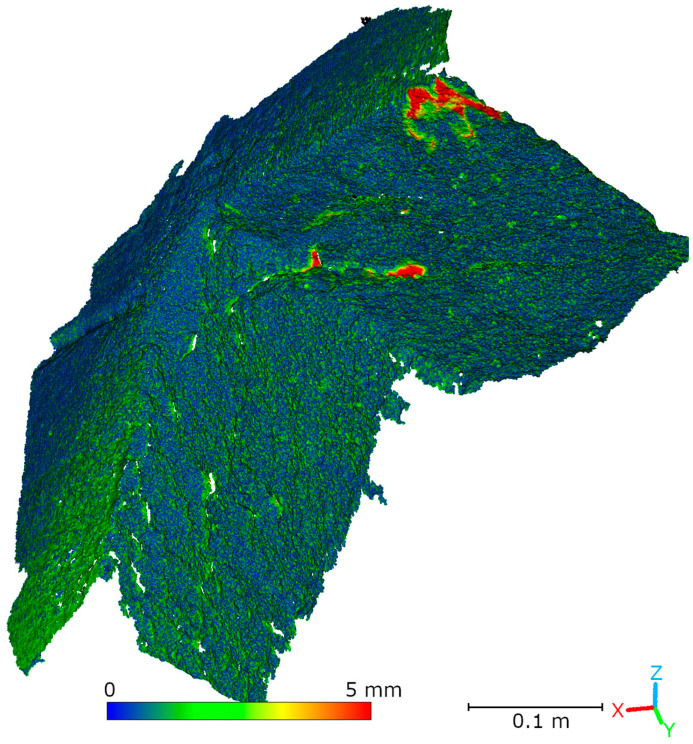
Comparison of TLS and HLS IR scan via cloud-to-cloud distance (C2C) for the common fragment.

To compare point clouds obtained using HLS and TLS, the data was registered to a common coordinate system. Registration was performed using the ICP algorithm in CloudCompare (v. 2.13.1) software. Afterward, the distances between the TLS reference cloud and the HLS clouds were calculated. Figure 18 and Table 3 show the registration results for the Blue mode data. For data acquired in IR mode, two variants of the analysis are presented. The first, shown in Figure 19 and Table 4, covers the entire cloud registered in IR mode. However, note that much of this data comes from an area not measured by the TLS scanner due to limited visibility. Therefore, a second analysis was performed that took into account only the part of the IR cloud that overlapped with the TLS data. The results of this comparison are presented in Figure 20 and Table 5. The analysis of the distance distribution between the TLS and HLS clouds revealed good agreement between both methods and the reference data for the common fragment. In IR mode, 89.6% of the points were within ±1.5 mm, in Blue mode, it was 89.2% (R1-C2, R2-C3, R2-C4).

For the analysis, an area encompassing the measured defect was extracted from the three point clouds. It should be noted that the TLS dataset was previously resampled to 2 mm spacing to standardize the data resolution across all datasets and improve computational efficiency during subsequent processing. Although this reduced the original data density, it ensured consistency in subsequent analyses (Surface Variation). Data density analysis revealed that the TLS point cloud contained significantly fewer points per unit area than the HLS point clouds. The TLS point cloud contained 3582 points after resampling (9466 points in the original data). The HLS Blue mode point cloud contained 4,342,859, while IR mode contained 535,909 points for an area of approximately 0.10 m^2^. This high data density enables highly accurate analysis of local damage (R2-C5, R1-C8).

**Figure 21:**
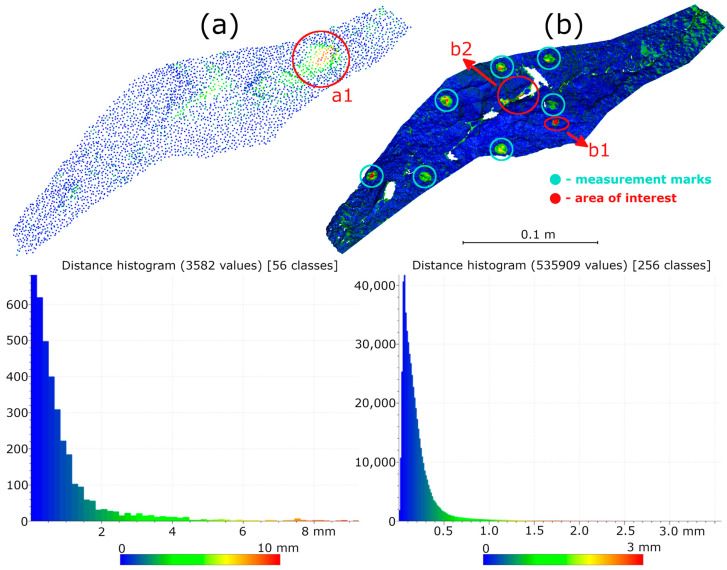
Distances between point clouds: (**a**) HLS Blue mode—TLS; (**b**) HLS Blue mode—HLS IR mode.

To further evaluate the consistency between scanning modes distances were calculated between the reference (Blue) and test point clouds (IR and TLS). The graphical results of the comparison are shown in Figure 21.

For TLS, the average distance to the reference point cloud was ±0.8 mm. The standard deviation of the errors was 1.1 mm. Assuming a normal distribution and a 95% confidence level, the confidence interval for the mean error was calculated to be 0.80–0.87 mm (R2-C1-R2). The analysis showed that 90% of points were within ±2 mm and 70% within ±1 mm. According to the device specifications (≤1 mm ± 10 ppm/m), and considering that the distance between the analyzed area and the nearest TLS station was approximately two meters, the expected positional accuracy for individual TLS points in this region is around 1.02 mm (R2-C6). It is worth noting that the obtained confidence interval falls within the declared accuracy range of the TLS. This means that the observed differences (an average of 0.8 mm) do not exceed the device’s expected measurement uncertainty. Therefore, these differences cannot be interpreted as clear geometric differences between the systems, but rather as being consistent with the TLS’s accuracy range (R2-C1-R2). For the IR point cloud, the average distance was ±0.2 mm, with 90% of points within ±0.37 mm. In Figure 21 three areas of interest and the locations of measurement markers are highlighted. The areas of interest are regions with the largest differences between point clouds. The first area, labeled a1, is on the TLS point cloud. Due to the one-month time gap between measurements, this area showed increased degradation. A small section of the retaining wall was damaged, indicating progressive deterioration. Apart from this section, the data aligned with an accuracy of approximately ±1 mm. The second area, labeled b1, includes minor surface damage (approximately 5 mm), likely a piece of coarse mortar or plaster accidentally removed during measurement setup reorganization. This fragment is visible in the IR data but absent in the later Blue mode measurement. The area labeled b2 encompasses a defect in form of a crack in the retaining wall. The difference analysis showed that blue light scanning captured a slightly larger portion of the crack than infrared scanning, possibly indicating greater resilience of blue light technology to varying lighting conditions. However, comparing both scans to the image in Figure 7, it was noted that the deepest part of the crack was not fully captured in HLS measurement. This was due to excessive lighting contrast, which limited the scanner’s ability to register dimly lit elements.

It should be emphasized that differences in areas marked as “measurement markers” are not measurement errors. The first measurement (IR mode) did not require markers. Markers were placed near the crack only before the Blue mode scan. Differences in the difference raster, on the order of approximately 2 mm in these areas, are thus a result of the change in measurement methodology.

**Figure 22:**
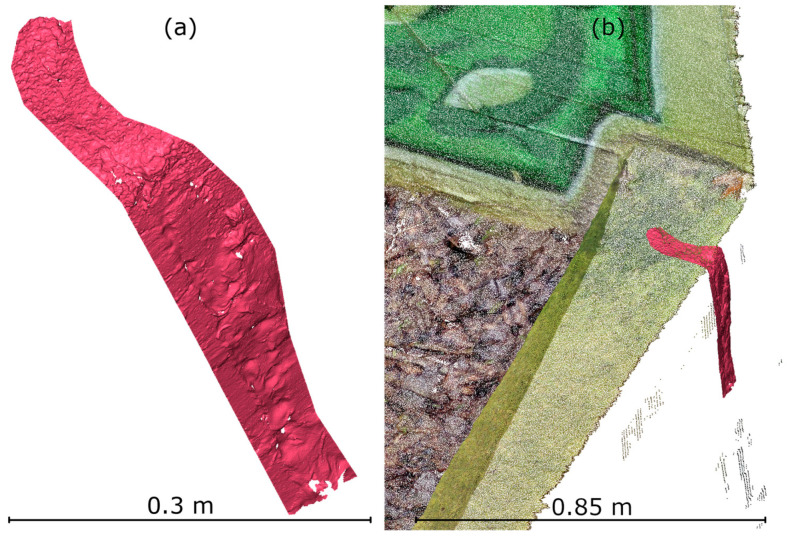
Second part of the crack measured in IR HLS mode. (**a**) View of the point cloud from the measurement. (**b**) HLS IR mode point cloud overlaid on TLS data.

In IR mode, a larger area was measured (due to the lack of need for measurement markers in hard-to-reach locations). An additional crack was identified, shown in Figure 22a. Data analysis revealed that this area was not captured in the TLS point cloud, as it was in a blind spot, as shown in Figure 22b.

**Figure 23:**
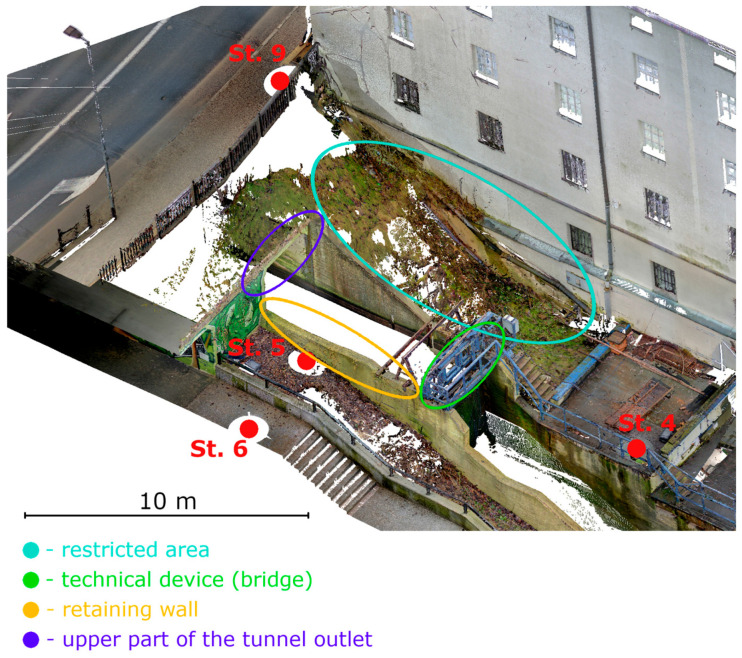
Layout of selected measurement stations. Colored outlines correspond to the areas shown in the legend. Red points mark measurement stations.

This was caused by the arrangement of measurement stations. Location of all stations are presented in Figure 23. Station 4 was positioned too low, causing a structural element impounding water in the intake chamber to obscure the defect location. Stations 5 and 6 were located on the opposite side of the retaining wall forming the intake chamber. Station 9 was relatively well-positioned, but part of the retaining wall was obscured by the upper part of the tunnel outlet, making only half of the wall near the bridge visible. The scanner stations were designed this way due to legal restrictions preventing access to the area using blue ellipse on Figure 23.

**Figure 24:**
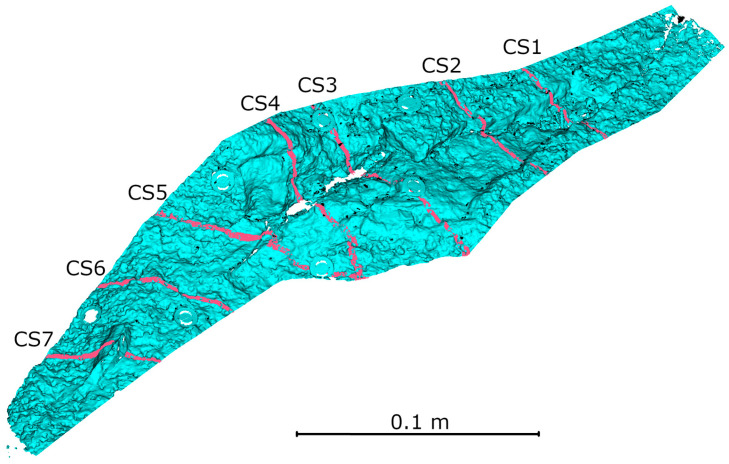
Position of cross-sections against the analyzed crack.

**Figure 25:**
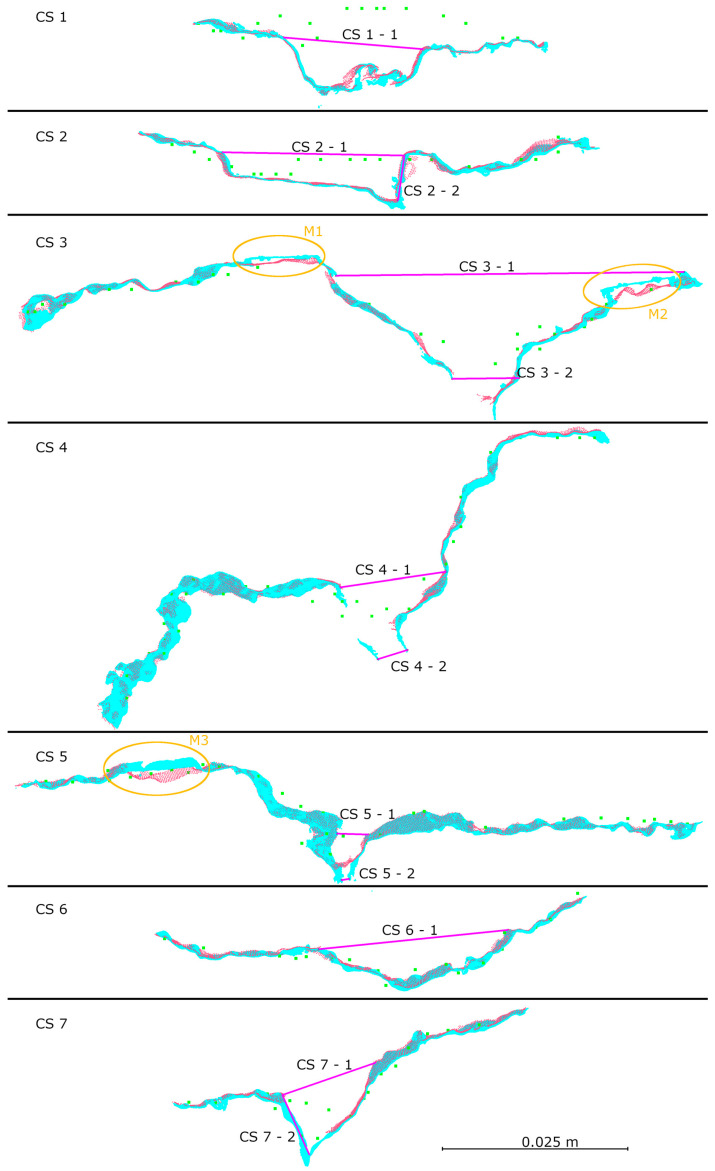
Cross-sections: green—TLS, red—HLS IR mode, blue—HLS Blue mode.

To compare the interpretive capabilities of each measurement method, seven cross-sections were made along the smaller defect. The position of the cross-sections relative to the crack, along with their numbering, is shown in Figure 24. The resulting cross-sections are shown in Figure 25, and the measurement results are presented in Table 6.

Based on the cross-sections presented in Figure 25 and the defect dimensioning data summarized in Table 6, a very high geometric consistency was observed between the HLS data in IR and Blue modes. The differences between these datasets for each cross-section did not exceed ±0.6 mm, and in most cases were ≤0.3 mm, confirming the high repeatability of measurements regardless of the device’s operating mode. Table 6 includes 12 measurement pairs, with three cases (CS4-2, CS5-2, CS7-2) where measurement in IR mode was not possible due to excessive lighting contrast. The average difference between IR and Blue from both laboratory and field measurements was ±0.24 mm. The largest measured crack width was recorded in cross-section CS3, at 47.5 mm, while the remaining defect widths ranged from 4 to 25 mm. In cross-sections CS3 and CS5, areas M1, M2, and M3 were marked. These are locations where differences between the data from different modes were observed. Analysis of these fragments in relation to the entire section indicates that these are locations where measurement markers were placed (see Figure 21), and thus are not the result of potential measurement errors.

**Figure 26:**
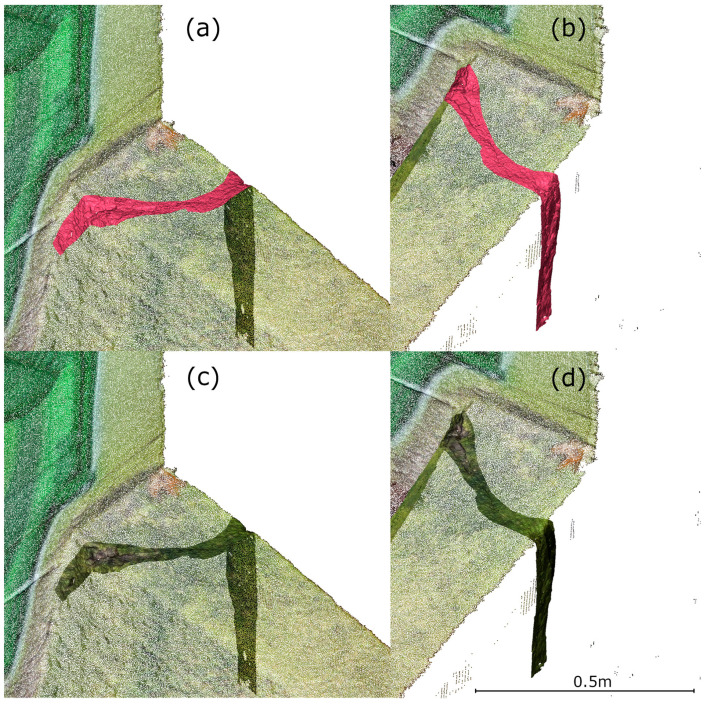
Integrated point clouds: (**a**) northern view—original colors; (**b**) northwestern view—original colors; (**c**) northern view—interpolated colors, (**d**) northwestern view—interpolated colors.

Following the accuracy analyses, it was decided to integrate the IR HLS scan data with the TLS data. The choice of IR data was based on two criteria: first, the analyses showed that the accuracy of the IR scan is comparable to the Blue scan, while generating fewer points, which prevents data overrepresentation in the crack area and reduces the volume of the resulting point cloud. Second, the lack of need for measurement markers enables measurement in hard-to-reach areas where placing markers would be difficult or impossible. The TLS point cloud segment representing the area of interest was removed, and HLS data were incorporated in its place. The combined point cloud is shown in Figure 26.

Since HLS point clouds do not contain color information, two approaches were implemented during data integration. Integration without color—where the HLS point cloud visibly differs in color (a uniform color can be chosen but will be consistent for the entire segment). Integration with color interpolation—where HLS data were colored based on the TLS point cloud information. This resulted in a unified point cloud that accurately represents both the crack geometry and its spatial relationship to the entire object, while preserving color information.

## 5. Discussion

The analyses demonstrated that the high precision and data density of HLS enable accurate representation of defect geometry and precise dimensioning. In laboratory conditions, registration accuracy was approximately 0.5 mm for both HLS modes. In field conditions, however, it reached about 1.0 mm due to less favorable scanning geometry. Laboratory measurements were taken with the scanner operating at an optimal angle and close range (approximately 1.5 m), which significantly improved geometric consistency. In the field, achieving such alignment was not possible. Registration accuracy was satisfactory, confirming the feasibility of the proposed method.

Analyzing the cross-sectional measurements revealed three limitations that affected the accuracy of the IR mode. First, the IR mode could not detect cracks smaller than ~2 mm and captured only slight deformations, which were insufficient for reliable measurements. It is not possible to determine the exact threshold width at which this limitation no longer applies, as the dataset does not include enough defects in the 2–5 mm range. However, cracks wider than 5 mm (e.g., LC2-2, LC2-8) were clearly identifiable and measurable. Second, IR mode produced noisy measurements in internal corners with angles close to 90°, which made it difficult to extract clean cross-sectional profiles. This phenomenon was confirmed in the laboratory (e.g., LC1-4, LC2-6). Without Blue mode data, it was impossible to distinguish whether the geometry reflected an actual feature or scanning noise. Third, the IR mode had limited ability to penetrate deep cracks under variable lighting conditions. It mostly captured the outer opening of cracks but failed to register internal surfaces. This was evident in both field and laboratory settings, highlighting a key limitation of this mode.

Based on these observations, Blue Mode appears to be the more appropriate tool for measuring cracks. Although it requires the use of measurement markers, its higher spatial resolution and interpretability allow for more reliable and complete measurements. The need for markers is offset by the enhanced ability to map internal geometry, as demonstrated by the analysis of Laboratory Crack 1 (Figure 13). While less precise, the IR mode remains a valuable tool in some scenarios. It successfully supplemented the TLS dataset by filling blind spots. Additionally, this mode may be suitable for different types of defects, such as wide and shallow surface losses without abrupt geometric changes or obstructions (R1-C6).

Tests conducted on reference models with known dimensions (2.5, 5, and 7.5 mm) confirmed that the Blue mode can reproduce crack geometry with an accuracy of ±0.2 mm. Analysis of the results showed good agreement with nominal values, as well as high measurement repeatability, as evidenced by low standard deviations (0.04–0.05 mm). In engineering applications such as structural damage diagnostics, these results suggest that the HLS scanner in Blue mode can accurately document crack geometry. Similar observations regarding the ability to map narrow cracks using structured light technology have been reported in earlier studies [36].

During measurement setup reorganization, a small piece of coarse plaster, approximately 5 mm in diameter, was accidentally removed. This minor change was visible in the model created from the HLS data, allowing for the assessment of the defect’s size.

When performing HLS measurements, attention must be paid to the object’s lighting differences. The structured light technology used by HLS is sensitive to this phenomenon. Excessive illumination or significant shadowing of certain object parts can prevent data acquisition for those areas. This is a known limitation of such devices [37,38,39]. A second significant limitation of HLS technology is the requirement of direct access to the examined object. The typical scanning distance is approximately 30 cm, meaning the operator must be able to physically bring the scanner close to the analyzed surface. This factor was a significant limitation in the analyses presented. Due to a lack of safe access, defects 1 and 2 could not be measured (see Figure 17) (R1-C3).

While TLS was not sufficient to fully capture the detailed crack geometry, it remains appropriate for global structural monitoring and provides valuable baseline data for long-term diagnostics (R1-C8). Note that the average distance between the HLS (Blue mode) and TLS clouds of 0.8 mm with a confidence interval of 0.80–0.87 mm reflects the TLS device’s nominal accuracy of approximately 1 mm (R2-C1-R2). The conducted research highlighted two main benefits of integrating TLS and HLS data. The first is the ability to precisely map local damage while maintaining spatial relationships with the entire building structure. This enables comprehensive documentation of both the overall geometry of the structure and specific defects critical for assessing its technical condition. The second benefit is the ability to address measurement gaps in TLS caused by geometric constraints or limited field access. Previously, such tasks were primarily accomplished through the integration of TLS with photogrammetry [15,16,17]. The obtained analysis results indicate significant potential for using HLS as an alternative or complementary source of spatial data in civil engineering, particularly for inspections and monitoring of technical conditions. The approach used in this study, integrating TLS and HLS data, enabled accurate mapping of the entire object’s geometry and detailed documentation of local damage that was not fully captured using TLS alone. The integration of data from different sources filled blind spots and improved the accuracy of defect analysis for the studied structure. The analysis of the performed cross-sections (Figure 25) demonstrated high consistency of results obtained in both HLS modes (IR and Blue), with differences ranging between 0.3 and 0.6 mm, depending on scanning mode and defect geometry. The research results confirm the validity of implementing modern 3D scanning methods for the continuous monitoring of infrastructure. As noted by Kozlov and Yurchenko [31], delayed recognition of structure degradation can lead to catastrophic failures. Meanwhile, Li et al. [32] emphasize the role of continuous geometry-based monitoring as a key factor in enabling predictive risk management.

The conducted research revealed progressive degradation of the SHP, indicating the need for regular geometric inspections of structures. The integration of TLS and HLS enables not only accurate documentation of damage but also informed engineering decisions regarding maintenance, repairs, or temporary decommissioning. Although this study focused on a small hydropower facility, the methods used could be applied to broader areas such as building diagnostics, infrastructure inspection, and conservation. This is especially true when combining global and local geometry is necessary (R1-C10).

## 6. Conclusions

Based on the conducted analyses, the following conclusions can be drawn:

- Integration of TLS and HLS data enables accurate representation of both the overall geometry of the object and its local damage.
- HLS, particularly in IR mode, allows effective measurement of hard-to-reach areas without the need for measurement markers.
- HLS data can effectively fill TLS measurement gaps caused by object geometry or limited field access.
- Accuracy analysis showed high consistency of HLS data, with differences between the two available modes not exceeding 0.37 mm, confirming the device’s effectiveness in both modes.
- Differences between HLS and TLS data were ≤2 mm for 90% of points and ≤1 mm for 70% of points.
- Cross-sectional analysis of the crack enables assessment of damage geometry with submillimeter accuracy. Both HLS modes provide consistent results, with Blue mode being more effective in capturing deep cracks under varying lighting conditions.
- High lighting contrast significantly hinders HLS measurements.
- The hybrid approach (TLS + HLS) shows significant potential for inspections and monitoring of the technical condition of civil engineering facilities like hydraulic structures.

## Figures and Tables

**Table 1:** Accuracy and repeatability of HLS measurements (Blue mode) for nominal defect widths.

Nominal Width (mm)	Iteration (mm)			Mean (mm)	Difference (mm)	Std. (mm)
	I	II	III			
2.5	2.64	2.63	2.75	2.67	0.17	0.05
5	5.16	5.08	5.09	5.11	0.11	0.04
7.5	7.75	7.76	7.64	7.72	0.22	0.05

**Table 2:** Results of laboratory cross-sections measurement.

Cross Section	HLS IR [mm]	HLS Blue [mm]	Difference [mm]
LC1-1	8.4	8	−0.4
LC1-2	m.n.p.	1.4	m.n.p.
LC1-3	6.4	6.2	−0.2
LC1-4	m.n.p.	5.9	m.n.p.
LC1-5	m.n.p.	1.7	m.n.p.
LC1-6	6.1	6.4	0.3
LC2-1	7	6.7	−0.3
LC2-2	5.2	5.1	−0.1
LC2-3	m.n.p.	1.4	m.n.p.
LC2-4	m.n.p.	1.5	m.n.p.
LC2-5	7.3	7.3	0
LC2-6	6.2	6	−0.2
LC2-7	m.n.p.	1.6	m.n.p.
LC2-8	5.7	6	0.3
LC2-9	6.8	6.3	−0.5
LC2-10	10.3	10.4	0.1

m.n.p.—measurement not possible.

**Table 3:** Distribution of cloud-to-cloud distances between TLS and HLS Blue point clouds.

Range [mm]	<0.5	0.5–1.0	1.0–1.5	1.5–2.0	2.0–2.5	2.5–3.0	3.0–3.5	3.5–4.0	4.0–4.5	>4.5
Points within range	13.4%	50.9%	25.0%	4.8%	1.4%	0.7%	0.5%	0.4%	0.3%	2.8%
Cumulative percentage of points	13.4%	64.3%	89.2%	94.0%	95.4%	96.1%	96.6%	97.0%	97.2%	100.0%

**Table 4:** Distribution of cloud-to-cloud distances between TLS and HLS IR point clouds.

Range [mm]	<0.5	0.5–1.0	1.0–1.5	1.5–2.0	2.0–2.5	2.5–3.0	3.0–3.5	3.5–4.0	4.0–4.5	>4.5
Points within range	8.1%	33.4%	19.4%	4.4%	1.1%	0.4%	0.3%	0.2%	0.2%	32.4%
Cumulative percentage of points	8.1%	41.5%	60.9%	65.3%	66.4%	66.8%	67.1%	67.4%	67.6%	100.0%

**Table 5:** Distribution of cloud-to-cloud distances between TLS and HLS IR point clouds for the common fragment.

Range [mm]	<0.5	0.5–1.0	1.0–1.5	1.5–2.0	2.0–2.5	2.5–3.0	3.0–3.5	3.5–4.0	4.0–4.5	>4.5
Points within range	12.0%	49.3%	28.3%	6.2%	1.7%	0.6%	0.4%	0.3%	0.2%	1.0%
Cumulative percentage of points	12.0%	61.3%	89.6%	95.8%	97.4%	98.0%	98.4%	98.7%	99.0%	100.0%

**Table 6:** Measurement results of cross-sections.

Cross Section	Measurement	HLS IR [mm]	HLS Blue [mm]	Difference [mm]
CS 1	CS 1-1	18.7	18.9	0.3
CS 2	CS 2-1	24.2	24.5	0.3
	CS 2-2	6.2	6.2	0.0
CS 3	CS 3-1	47.5	46.9	−0.6
	CS 3-2	8.8	9.0	0.2
CS 4	CS 4-1	14.1	14.5	0.4
	CS 4-2	m.n.p.	4.1	m.n.p.
CS 5	CS 5-1	4.0	4.2	0.1
	CS 5-2	m.n.p.	1.5	m.n.p.
CS 6	CS 6-1	25.5	25.6	0.1
CS 7	CS 7-1	13.2	13.3	0.2
	CS 7-2	m.n.p.	8.8	m.n.p.

m.n.p.—measurement not possible.

## Data Availability

The original contributions presented in this study are included in the article. Further inquiries can be directed to the corresponding author.
